# Role of FGF21 and Leptin for the Diagnosis of Metabolic Health in Children with and without Obesity

**DOI:** 10.3390/jpm13121680

**Published:** 2023-12-03

**Authors:** Eleni M. Domouzoglou, Antonios P. Vlahos, Michail I. Papafaklis, Vasileios K. Cholevas, Nikolaos Chaliasos, Ekaterini Siomou, Lampros K. Michalis, Agathocles Tsatsoulis, Katerina K. Naka

**Affiliations:** 1 Child Health Department, Faculty of Medicine, School of Health Sciences, University of Ioannina, Stavrou Niarchou, 45110 Ioannina, Greece; 2 Second Department of Cardiology, Faculty of Medicine, School of Health Sciences, University of Ioannina, Stavrou Niarchou, 45110 Ioannina, Greece; m.papafaklis@yahoo.com (M.I.P.); anaka@uoi.gr (K.K.N.); 3 Department of Endocrinology, Faculty of Medicine, School of Health Sciences, University of Ioannina, Stavrou Niarchou, 45110 Ioannina, Greece

**Keywords:** obesity, overweight, fibroblast growth factor 21, leptin, metabolism, adiponectin, flow-mediated dilation

## Abstract

Obesity and unfavorable metabolic profiles increase the risk for cardiovascular complications in adults. Although it is important to distinguish different metabolic health states at an early stage, there are limited data on the related value of biomarkers in childhood. We aimed to identify biomarkers for the detection of different metabolic health states in children with and without obesity. The serum levels of metabolic regulators (fibroblast growth factor 21 [FGF21], leptin, adiponectin and insulin-like growth factor binding protein 1) and vascular indices (flow-mediated dilation [FMD] and carotid intima-media thickness) were assessed in 78 children. Differences between the metabolically healthy and unhealthy state within children with normal weight (MHN vs. MUN), and within children with overweight/obesity (MHO vs. MUO) were investigated; the discriminatory power of the biomarkers was studied. Both MUN and MUO groups expressed altered lipid and glucose homeostasis compared to their healthy counterparts. The metabolic unhealthy state in children with normal weight was linked to higher FGF21 levels which had good discriminatory ability (area under the curve [AUC]: 0.71, 95% CI: 0.54–0.88; *p* = 0.044). In overweight/obese children, leptin was increased in the metabolically unhealthy subgroup (AUC: 0.81, 95% CI: 0.68–0.95; *p* = 0.01). There was a decrease in FMD indicating worse endothelial function in overweight/obese children versus those with normal weight. Distinct states of metabolic health exist in both children with normal weight and overweight/obese children. FGF21 and leptin may help to identify the metabolic unhealthy state in children with normal weight and in overweight/obese children, respectively, early in life.

## 1. Introduction

The complexity and increasing global dimensions of obesity, concerning adults, adolescents and children constitute a pandemic. Obesity has been associated with cardiovascular disease risk factors such as elevated blood pressure, altered lipid profile and glucose homeostasis, frequently coupled with increased carotid intima media thickness (cIMT) which may progress if not prevented [1,2]. Additionally, it is becoming increasingly important to distinguish between a metabolically healthy and unhealthy form of obesity [3,4]. Metabolically benign or healthy obesity is not accompanied by insulin resistance and early atherosclerosis as assessed by cIMT in adults [5]; this type of obesity may represent as much as 10–30% of the population with obesity [6]. It has also been suggested that the optimal treatment of childhood obesity should aim in improving the metabolically healthy state for increased benefit [7].

Metabolic health is also important in people without obesity, as increasing evidence supports that metabolic deregulations may lead to disease independently of the presence of obesity. Recent data raise the alarm about the underestimated prevalence of metabolic-associated fatty liver disease in people who have normal weight [8]. Furthermore, unfavorable cardiovascular outcomes, i.e., increased risk of incident heart failure, cardiovascular disease–related mortality, have been described in metabolically unhealthy subjects of normal weight paralleled to outcomes seen in people with obesity [9]. Consequently, it would be highly enlightening to identify markers that may be able to distinguish between different metabolic states in subjects both with and without obesity, especially at an early stage, before the occurrence of irreversible metabolic and cardiovascular complications.

A few recent studies have indicated that certain indices (e.g., homeostatic model assessment for insulin resistance [HOMA-IR], waist–height ratio, serum uric acid, BMI) as well as male gender are associated with the presence of metabolically unhealthy obesity and its cardiometabolic complications in children [10,11]. However, there is a dearth of evidence on serum biomarkers which could help discriminate the metabolic health state in children with and without obesity.

Our primary aim was to study the value of important metabolic regulators implicated in obesity and insulin resistance (fibroblast growth factor 21 [FGF21], leptin, adiponectin and insulin-like growth factor binding protein 1 [IGFBP1]) as markers of metabolic health in overweight/obese children and those with normal weight. Additionally, we investigated the association of the vascular indices of endothelial function and early atherosclerosis, i.e., flow mediated dilation (FMD) and cIMT, with obesity and metabolic health.

## 2. Materials and Methods

### 2.1. Participants and Study Protocol

Seventy-eight healthy children (42 boys and 36 girls), aged 8 to 16 years, were included in this cross-sectional study. All children were free of any disease and attended the Pediatric Department of the University Hospital for routine examination prior to initiation of sports activities. The study was conducted according to the Declaration of Helsinki and written informed consent was signed by the parents/guardians. The study protocol was approved by the local Bioethics Committee.

All data were prospectively collected. Personal history, demographic and anthropometric data were recorded, and blood sampling was performed at 8 am after an overnight fast. Standing height was measured to the nearest 0.1 cm on a wall-mounted stadiometer. Body weight was measured in the morning, with light clothing and no shoes on, using an electronic scale. Waist and hip circumferences were defined based on the IDF criteria and the waist to hip ratio was calculated [12]. The BMI was calculated as weight/height^2^ (kg/m^2^). Systolic (SBP) and diastolic (DBP) blood pressure was assessed with an electronic sphygmomanometer at rest before any further procedures were initiated, in the morning after overnight fasting and in a calm and quiet environment.

The metabolically unhealthy state was defined according to the following criteria [13]: HOMA-IR ≥ 3, triglycerides (TRG) ≥ 1.243 mmol/L, high-density lipoprotein cholesterol (HDL) ≤ 1.036 mmol/L, fasting blood glucose ≥ 5.55 mmol/L, SBP ≥ 130 mmHg and/or DBP ≥ 85 mmHg. Four groups were formed based on the BMI and the presence of the above criteria for metabolically unhealthy status as follows: (1) children with normal weight without any of the above criteria (i.e., metabolically healthy [MHN]), (2) children with normal weight with at least one of the criteria (metabolically unhealthy [MUN]), (3) children with obesity without any of the above criteria (MHO), (4) children with obesity with at least one of the above criteria (MUO).

### 2.2. Serum and Imaging Analyses

#### 2.2.1. Serum Analysis

Serum samples were collected after a standard centrifuge procedure, and they were either directly assessed or stored at −80 celsius degrees in a laboratory freezer until their assaying.

Serum glucose, total cholesterol (TChol), TRG, HDL and the transaminases aspartate aminotransferase (AST) and alanine aminotransferase (ALT) were analyzed on the Beckman Coulter AU5800 Clinical Chemistry (Brea, CA, USA). Low-density lipoprotein cholesterol (LDL) was derived from the Friedewald formula. Apolipoprotein A (apoA), apolipoprotein B (apoB) and lipoprotein a [Lp(a)] were analyzed on the Siemens BN ProSpec nephelometer (Erlangen, Germany). Free thyroxin (fT4), thyroid stimulating hormone (TSH) and insulin were determined by 3rd generation (HYPERsensitive hTSH) Access Immunoassays on a Beckman Coulter Dx1800 analyzer (Brea, CA, USA). HOMA-IR was calculated by the following formula: fasting blood glucose (mmol/L) × fasting blood insulin (μU/mL)/22.5 [14]. HbA1c was measured on a Bio-Rad Variant II, HPLC analyzer (Hercules, CA, USA). C reactive protein (CRP) was measured using turbidimetry, IMMAGE Immunochemistry Systems and Calibrator 5 Plus, Bechman Coulter (Brea, CA, USA).

The measurement of the biomarkers FGF21, adiponectin, leptin and IGFBP1 was carried out by enzyme-linked immune-assays (ELISA). The commercial kit (Human FGF21 Quantikine ELISA) from R&D Systems, Minneapolis, MN, USA) was used for FGF21. The sensitivity of the method was 4.67 pg/mL, and the coefficients of variation (CVs) were 3.9% and 10.9% for intra and inter assays, respectively. For leptin (Leptin Sandwich ELISA), the kit by DRG Instrs GmbH (Marburg, Germany) was used. The sensitivity of the method was 1.0 ng/mL and the CVs 6% and 9%, respectively. Adiponectin (Human adiponectin ELISA) was assessed using the kit by Biovendor (Research and Diagnostic Products, Brno, Czech Republic). The sensitivity of the method was 26 ng/mL, and the respective CVs were 5.9% and 6.3%. IGFBP1 was measured using the kit by DIA Source (IGFBP-1 ELISA KARME01; Louvain-la-Neuve, Belgium). The sensitivity of the method was 0.02 ng/mL and the respective CVs 5.2% and 5.9%.

#### 2.2.2. Ultrasound Studies

All studies were performed after fasting for 6 h and all measurements were taken in the supine position, in a quiet environment and in a temperature-controlled room (≈22 °C). For optimal imaging of the brachial and common carotid arteries, an echo-Doppler ultrasound (Ultrasound ATL, HDI 5000, Bothell, WA, USA) and a 5–12 MHz transducer were used.

FMD protocol: Endothelial function was assessed by measurement of endothelium-dependent vasodilation in the right brachial artery in response to hand hyperemia, based on previously described methodology according to recently published recommendations in children and adolescents [15,16]. Images were acquired at baseline and every 30 s, from the first to the third minute after deflation of a wrist cuff inflated to 250 mmHg for 4 min. Brachial artery blood flow was measured by continuous wave Doppler at baseline and 15 s after cuff release. FMD was calculated as the percent increase in arterial diameter during the first 3 min of hyperemia compared with the diameter at rest.

Shear stimulus and normalized FMD: Shear rate is an estimate of shear stress without viscosity and was used to quantify the stimulus for FMD. Shear rate was calculated as mean blood flow velocity/vessel diameter. The peak of the shear stress stimulus induced by reactive hyperemia occurs within the first seconds post cuff-release, and thus, the brachial artery mean blood flow velocity at 15 s post cuff-release was used for calculating the peak shear rate [17]. The magnitude of the imposed shear stress stimulus has great importance on influencing the magnitude of FMD [18]. Thus, to compare the FMD response between subjects and groups taking into account any variations in the magnitude of the shear stress stimulus, the FMD response was normalized to the magnitude of the shear stimulus [19]. For that purpose, the peak FMD during the first 90 s (i.e., within the time that the peak vessel diameter adaptation is typically observed) was divided by the peak shear rate.

cIMT protocol: Common carotid artery (CCA) IMT measurement was performed using a standardized protocol based on published recommendations in children and adolescents [15,16]. Three consecutive longitudinal images of each CCA 1–2 cm proximal to the bifurcation were acquired. Measurements were always made at the far wall of the artery. The mean value of IMT for right and left CCA was obtained by averaging the three measurements at each artery. Finally, the maximum cIMT of the two CCAs was determined.

Off-line analysis and measurement of brachial artery end-diastolic diameter and IMT were performed by two blinded operators using the QLAB software (version 5, Philips Ultrasound, Bothell, WA, USA).

### 2.3. Statistics

Most variables of interest (including FGF21, leptin, HOMA-IR, IGFBP1 and normalized FMD) were found to deviate significantly from the normal distribution (Kolmogorov–Smirnov test). Continuous variables are presented as medians [interquartile range (IQR)]. Non-parametric analysis with the Mann–Whitney U test was performed for comparing continuous variables between two groups. Receiver operating characteristic curve analysis was employed to identify the best cut-off values (Youden index: max [sensitivity and specificity]) of biomarkers for discriminating between the metabolically healthy and unhealthy state within the groups of children with normal weight and overweight/obese children. The level of significance was set at *p* < 0.05. The SPSS statistical software package (version 21.0 for Windows, IBM Corp., Armonk, NY, USA) was used for the analysis.

## 3. Results

### 3.1. Metabolic Health State in Children with Normal Weight and Children with Obesity

**Figure 1:**
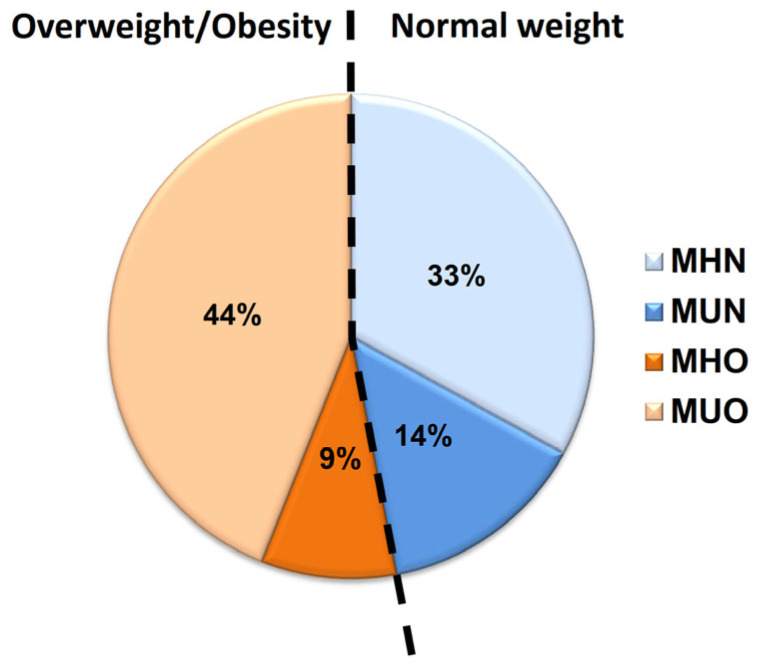
Childrens’ groups based on the body mass index and the presence of metabolically unhealthy status. Subgroups based on body mass index (normal weight vs. overweight/obese) and the presence of criteria for metabolically unhealthy status. Percentages are calculated based on the total number of children included in our study. MHN: metabolically healthy normal weight; MUN: metabolically unhealthy normal weight; MHO: metabolically healthy overweight/obesity; MUO: metabolically unhealthy overweight/obesity.

Of the 78 children included in the study, 41 (53%; 19 boys and 22 girls) had increased body weight (overweight or obesity), and the remaining 37 (47%; 23 boys and 14 girls) had normal weight for their age and sex (Figure 1). Within the group of children with normal weight, we identified 11 (30%; 14% of all children) that met criteria for the metabolically unhealthy state. Within the group of overweight/obese children, we identified 7 (17%; 9% of all children) that lacked criteria for the metabolically unhealthy state, and thus were categorized as metabolically healthy.

Comparative baseline characteristics/somatometrics, serum biochemistry of glucose homeostasis, hormonal regulation and the lipid profile in the subgroups with normal weight and overweight/obesity based on the metabolic health are shown in Table 1 and Table 2, respectively. Children with obesity in the metabolically unhealthy subgroup were overall older compared to the MHO. Glucose homeostasis was more prominently affected in the normal weight unhealthy versus the healthy group (higher glucose, insulin and HOMA-IR in MUN), while within the group with obesity the deregulation influenced both glucose (higher insulin and HOMA-IR in MUO) and lipid (lower HDL and higher TRG in MUO) metabolism. Additionally, in children with normal weight, TSH levels were higher while fT4 levels tended to be lower in the metabolically unhealthy state.

### 3.2. Biomolecules as Markers of Metabolic Health State

The biomarker levels (FGF21, leptin, IGFBP1 and adiponectin) in the different metabolic subgroups are shown in Table 3. FGF21 was the only molecule with significantly different regulation in the MUN group compared to the MHN group by a 3-fold increase (86.2 vs. 28.7 pg/mL, respectively; *p* = 0.044), a finding which was also accompanied by a significant increase in the FGF21/adiponectin ratio. In the overweight/obese group, leptin was found to be significantly increased in the MUO group compared to the MHO (18.1 vs. 10.3 ng/mL, respectively; *p* = 0.01). The serum expression of adiponectin and IGFBP1 remained unchanged between the two metabolic health subgroups.

**Figure 2:**
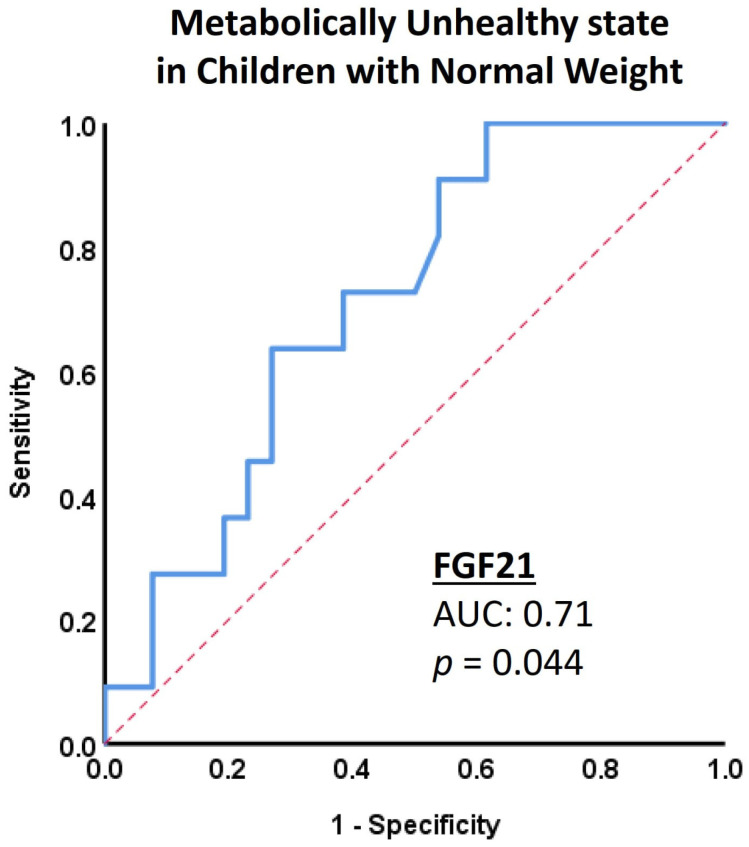
Diagnostic performance of FGF21 in children with normal weight. Receiver operating characteristic curve analysis for fibroblast growth factor 21 (FGF21) regarding the diagnosis of the metabolic unhealthy state within the group of children with normal weight shows good discrimination (area under the curve [AUC]: 0.71; 95% CI: 0.54–0.88).

**Figure 3:**
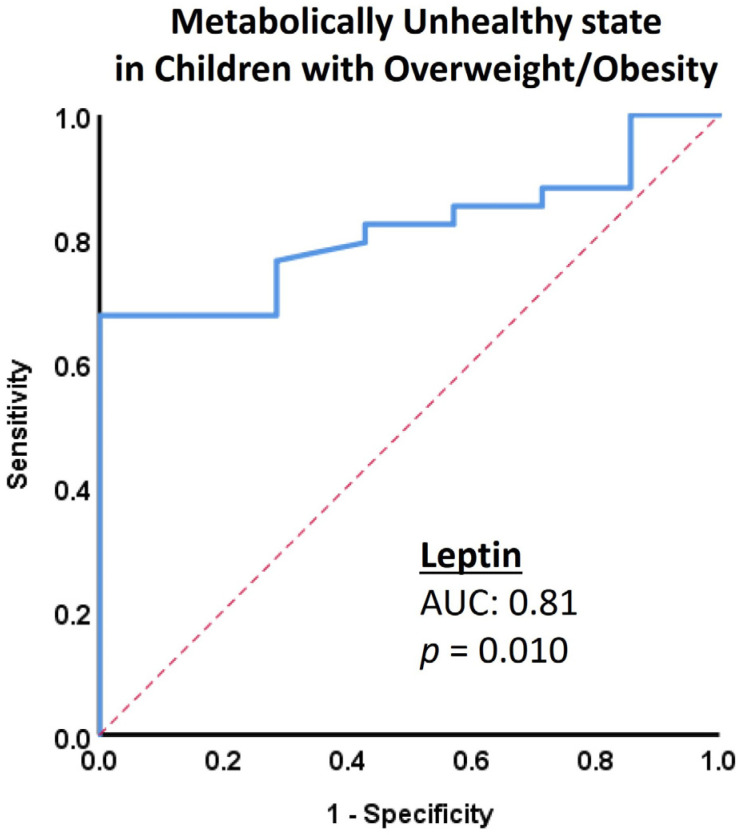
Diagnostic performance of leptin in overweight/obese children. Receiver operating characteristic curve analysis for leptin regarding the diagnosis of the metabolic unhealthy state within the group of overweight/obese children shows good discrimination (area under the curve [AUC]: 0.81; 95% CI: 0.68–0.95).

The ROC curve analyses for FGF21 and leptin to identify the metabolically unhealthy state within the group with normal weight and the overweight/obese group, respectively, are illustrated in Figure 2 and Figure 3. The areas under the curve for the two biomarkers showed good discriminatory ability both for FGF21 (0.71, 95% CI: 0.54–0.88; *p* = 0.044) and leptin (0.81, 95% CI: 0.68–0.95; *p* = 0.01) in the respective groups. The best cut-off value of FGF21 to identify the metabolically unhealthy state in children with normal weight was 17.05 pg/mL (sensitivity 100% and specificity 38.5%). In the case of overweight/obese children, the best cut-off value of leptin to identify the metabolically unhealthy state was 14.15 ng/mL (sensitivity 67.6% and specificity 100%).

### 3.3. Vascular Indices in the Different Metabolic States

Normalized FMD was significantly decreased in overweight/obese children compared to children with normal weight (0.06 [0.041–0.094] vs. 0.09 [0.045–0.125], *p* = 0.044), while there was no significant difference according to the metabolic health state within each of these groups (Table 1 and Table 2). There was no difference in the cIMT measurements between overweight/obese children and children with normal weight (0.47 [0.43–0.49] vs. 0.45 [0.42–0.48] mm, respectively; *p* = 0.264) as well as according to the metabolic health state within these groups (Table 1 and Table 2).

## 4. Discussion

Our study provides additional data supporting that different metabolic health states exist in individuals with obesity and, in particular, early in life in children/adolescents. Furthermore, we have shown that the metabolically healthy and unhealthy state may exist independently from the presence of obesity. Most importantly, we have identified FGF21 and leptin as significant biomarkers for the presence of the metabolically unhealthy state in children with normal weight and overweight/obese children, respectively. Lastly, our FMD results showed worse endothelial function in children with obesity compared to children with normal weight, but there was no significant difference in vascular function or structure according to the metabolic health state within the groups with overweight/obesity and normal weight.

In both our main groups (normal weight and overweight/obesity), the metabolic state (unhealthy vs. healthy) was linked to alterations in glucose and lipid metabolism. In overweight/obese children, there was a combination of low HDL and high TRG levels which is known to promote atherogenesis [20]. In children with normal weight, the only significant finding regarding lipids in the metabolically unhealthy group, was the increase in the ApoB/ApoA ratio without any significant difference in the HDL or the TRG levels. ApoB is known to be a sensitive marker of atherosclerosis and its serum levels are increased early, before the appearance of any other lipid dysregulation [21]. Our results support that the ApoB/ApoA ratio is sensitive to early and subtle changes in these lipoproteins, even in the absence of significant variation in their serum levels.

A major finding of our study is the significantly increased FGF21 serum levels differentiating the metabolically unhealthy group within children with normal weight. FGF21 is a metabolic regulator found to be secreted principally by the liver but also found in other tissues in lesser amounts [22]. FGF21 plays a significant role in metabolic homeostasis including the lipid profile, body weight and insulin sensitivity [23]. FGF21 serum levels are associated with the levels of apolipoprotein B and A and triglycerides [24]. Moreover, it has been described that FGF21 inhibits ApoA expression in human hepatoma cell line HepG2 via the FGF21-ERK1/2-Elk-1 pathway indicating the involvement of this molecule in the metabolic pathway of lipoproteins [25]. The role of FGF21 in this pathway is not clear and may represent a protective attempt towards the hepatic tissue, since ApoA is known to remove excess cholesterol molecules from the tissues and transport them within the HDL molecule towards the liver [26]. FGF21 has been shown to protect hepatocytes from injury and has diverse anti-fibrotic effects [27]. We also observed an increase in central thyroid regulation by TSH in the NMU compared to the NMH group; this increase coupled to the increased FGF21 levels is consistent with previous experimental findings of the mutual regulatory relationship between FGF21 serum levels and thyroid function and their possible link in regulating metabolism [28].

Of the biomarkers studied in children with normal weight, FGF21 was the only one that was found to be significantly increased in the serum of the metabolically unhealthy subgroup compared to the healthy subgroup, thereby showing the ability of FGF21 to detect subtle differences in an otherwise normal group of children. Hence, in a healthy phenotype which is less likely to be suspect for a metabolic fault, FGF21 performed as a serum marker of an unhealthy metabolic state, by increased expression of circulating levels. In contrast, FGF21 did not differ between the metabolic health states in children with obesity in our study. This is consistent with latest data in abdominally obese adults which did not show evidence of association between fasting plasma FGF21 and the metabolic health state but suggested that dynamic changes in circulating FGF21 levels in response to a nutritional challenge may reflect metabolic health status [29].

Within the group of overweight/obese children, the metabolic deregulation involved both glucose and lipid pathways linked to higher circulating levels of leptin. This hormone is secreted by adipose tissue, it is known to have an anorexigenic action and to regulate satiety, and its levels are increased proportionally to fat mass [30]. Leptin levels have also been shown to be increased in states of persistent hyperinsulinemia and insulin resistance [31]. This partially explains the present finding of increased leptin levels in our MUO group linked to the increased HOMA-IR compared to the MHO group. In contrast, leptin was low and no different in the MUN and MHN groups, regardless of the presence of increased insulin resistance in the MUN versus the MHN group. This finding leads us to speculate that obesity is a prerequisite for the impact of insulin on leptin levels. The children with obesity categorized as metabolically healthy were younger in age (≈10 years old) than the ones categorized as metabolically unhealthy (>12 years old), which is consistent with literature findings [13,32]. We speculate that there may be an adaptive mechanism regulating the shift between healthy and unhealthy obesity in a growing organism. It is possible that a growing child gradually loses the ability to use fat depots which may then lead to a shift of the excess of fat towards a malignant form of obesity. This is consistent with studies in humans showing that genetics and epigenetic mechanisms influence the modality by which fat is stored in the tissues. There is a theory of “limited adiposity expandability” which describes the transformation of adipose tissue under persistent positive energy balance. There is a rapidly increasing process of the size of pre-existing adipocyte cells in subcutaneous adipose tissue in response to the increased lipid accumulation (hypertrophy of adipose tissue). Under certain conditions and in specific age limits, this process may be followed by hyperplasia, which represents the increase in the number of adipose tissue cells [33,34]. Age is apparently an important factor in the potential of adipose tissue expandability [35].

Additional insights could be drawn by observing the waist to hip ratio, the waist circumference and the liver enzymes in our children/adolescents. The waist to hip ratio and waist circumference are indices of visceral adiposity and central obesity which have been frequently linked to dyslipidemia and insulin resistance in adolescents and adults [36]. In our study, neither index was significantly different in the comparisons between the two metabolic states within the normal weight and overweight/obese groups. This represents an important difference between the metabolic syndrome and the metabolic health, as in children metabolic health may exist independently from central obesity in contrast to the metabolic syndrome. Importantly, waist circumference may not always reflect the accurate proportion of visceral obesity and may be unable to differentiate it from the local cutaneous fat [32]. There is need for further improvement in the identification of the “bad” fat based on both the presence of obesity and efficient metabolic markers. Liver enzymes increase in fatty liver disease [37]. In our study they were found to be within normal limits and did not differ between the groups. This suggests that the identification and early modification of these initial states of metabolic deregulation may offer an opportunity for successful prevention before structural changes develop.

To further assess the potential effect of the metabolically unhealthy state on vascular function and structure, we assessed FMD and cIMT in all our children. FMD was significantly decreased in children with obesity compared to children with normal weight. Therefore, FMD is shown to be a sensitive marker of vascular function in children and adolescents, and we have confirmed that obesity affects vascular health early in life. Previously reported study data are controversial on this matter; in a study of 35 children with obesity with a mean age of 9 years, no association was found between FMD and any clinical and biochemical characteristics, while another study of 252 children aged 9 to 18 years showed that FMD was positively associated with high adiposity [38,39]. As far as the metabolic health state is concerned in the overweight/obese and normal weight groups, we observed that there were no significant differences in FMD in our study. Overall, these findings indicate the predominant impact of obesity on early signs of cardiovascular disease in childhood, whereas the metabolic health state may have a secondary role.

The importance of maintaining a normal body weight remains a primary objective; this is also supported by a metabolomic pattern associated with a higher inherent cardiovascular risk in metabolically healthy obese adults, although they may not present increased cardiovascular events at 10 years [40]. However, discriminating between metabolic health states has also gained importance, and it would be of value to use sensitive markers for successfully detecting them in subjects with different body weights. Following further confirmatory studies, FGF21 and leptin could be included in a metabolic testing panel applied in screening models for routine check-up early in life, in children and adolescents. Such a screening strategy could help in planning more successful life-style modifications towards a combination of lower body weight and a healthier metabolic state. There is strong evidence supporting that metabolic health may be achieved early in life and maintained throughout adulthood by applying appropriate habits such as exercise and food intake [32,41].

Our study is limited by the sample size which was not large. Furthermore, this study was not designed to include a follow-up of the enrolled subjects, as we did not aim to investigate the predictive value of biomarkers for pathologic conditions in the future. However, our cross-sectional study showed for the first time that FGF21 and leptin were able to discriminate the metabolic state in children without and with obesity, respectively. The results of our exploratory analysis are hypothesis-generating and need to be investigated further in larger appropriately sized studies.

## 5. Conclusions

We have shown that different states of metabolic health exist, both in children with normal weight and in overweight/obese children. The metabolically unhealthy state is characterized by distinct dysregulations which are reflected by the levels of FGF21 and leptin. We propose FGF21 as a marker able to differentiate metabolic health in children with normal weight, while leptin could be used to detect unfavorable metabolic profiles under states of already established obesity. FMD may be an additional tool to assess vascular health in children. Further longitudinal studies are needed to prove the potential prognostic value of these markers in children and adolescents.

## Figures and Tables

**Table 1:** Somatometrics, laboratory investigations and vascular indices in the metabolically healthy (MHN) compared with the metabolically unhealthy (MUN) state within the group of children with normal weight.

Variable	MHN	MUN	*p* Value
Age (years)	13.1 (10.0–15.3)	13.1 (10.8–15.1)	0.62
BMI (kg/m^2^)	17.4 (16.5–20.3)	18.4 (17.1–23.0)	0.53
Waist circumference (cm)	65.5 (57.3–72.3)	70.0 (65.0–73.0)	0.28
Waist-Hip ratio	0.81 (0.77–0.86)	0.83 (0.78–0.86)	0.79
***Glucose Homeostasis***			
Glucose (mmol/L)	4.66 (4.33–5.05)	4.99 (4.60–5.49)	0.060
Insulin (μU/mL)	5.8 (4.3–7.4)	9.5 (5.6–18.5)	0.027
HOMA-IR	1.27 (0.89–1.6)	2.22 (1.14–4.10)	0.038
***Liver Enzymes***			
ALT (IU/L)	14.0 (10.5–18.5)	16.0 (12.0–18.0)	0.44
AST (IU/L)	21.0 (17.5–28.5)	22.0 (17.0–29.0)	0.90
***Thyroid Regulation***			
TSH (μIU/L)	1600 (1400–2400)	2500 (1800–2700)	0.027
fT4 (pmol/L)	11.33 (10.42–12.87)	10.29 (9.65–11.97)	0.074
***Lipid Profile***			
TChol (mmol/L)	4.19 (3.40–5.01)	4.14 (3.49–4.71)	1.00
HDL (mmol/L)	1.37 (1.23–1.49)	1.16 (1.01–1.48)	0.077
TRG (mmol/L)	0.62 (0.45–0.76)	0.80 (0.49–1.26)	0.086
LDL (mmol/L)	2.51 (2.00–3.31)	2.67 (2.02–3.00)	0.72
Lp(α) (g/L)	0.11 (0.05–0.24)	0.08 (0.05–0.37)	1.00
ApoA (g/L)	1.45 (1.37–1.55)	1.36 (1.18–1.58)	0.21
ApoB (g/L)	0.67 (0.52–0.78)	0.79 (0.58–0.93)	0.10
ApoB/ApoA	0.44 (0.35–0.56)	0.54 (0.46–0.63)	0.041
***Vascular Indices***			
Normalized FMD	0.07 (0.04–0.12)	0.09 (0.04–0.22)	0.67
Carotid IMT (mm)	0.45 (0.43–0.48)	0.43 (0.38–0.49)	0.39

**Table 2:** Somatometrics, laboratory investigations and vascular indices in the metabolically healthy (MHO) compared with the metabolically unhealthy (MUO) state within the group of overweight/obese children.

Variable	MHO	MUO	*p* Value
Age (years)	9.9 (9.5–13.8)	12.4 (11.4–14.6)	0.048
BMI (kg/m^2^)	25.4 (21.1–29.1)	27.8 (24.7–30.0)	0.20
Waist circumference (cm)	88.0 (68.0–95.0)	87.8 (78.9–96.9)	0.37
Waist-Hip ratio	0.96 (0.79–0.98)	0.85 (0.82–0.92)	0.21
***Glucose Homeostasis***			
Glucose (mmol/L)	4.49 (4.10–4.88)	4.82 (4.38–5.13)	0.25
Insulin (μU/mL)	5.2 (3.9–9.2)	17.7 (10.4–28.3)	0.002
HOMA-IR	1.1 (0.8–2.0)	3.7 (2.1–6.5)	0.002
***Liver Enzymes***			
ALT (IU/L)	16.0 (10.0–26.0)	17.5 (12.8–28.3)	0.60
AST (IU/L)	21.0 (17.0–35.0)	22.0 (18.0–27.3)	0.78
***Thyroid Regulation***			
TSH (μIU/L)	2100 (1300–3000)	2000 (1500–2600)	0.70
fT4 (pmol/L)	11.58 (9.00–12.87)	10.29 (9.00–11.58)	0.44
***Lipid Profile***			
TChol (mmol/L)	4.84 (3.73–5.96)	4.22 (3.54–5.02)	0.43
HDL (mmol/L)	1.24 (1.09–1.5)	0.99 (0.89–1.26)	0.018
TRG (mmol/L)	0.59 (0.48–1.06)	1.46 (0.78–1.78)	0.007
LDL (mmol/L)	3.05 (2.33–4.17)	2.51 (1.82–3.25)	0.28
Lp(α) (g/L)	0.07 (0.05–0.09)	0.05 (0.02–0.19)	0.67
ApoA (g/L)	1.42 (1.29–1.55)	1.29 (1.14–1.41)	0.097
ApoB (g/L)	0.70 (0.62–0.97)	0.72 (0.58–0.96)	0.94
ApoB/ApoA	0.49 (0.47– 0.73)	0.66 (0.40–0.75)	0.49
***Vascular Indices***			
Normalized FMD	0.07 (0.05–0.11)	0.06 (0.04–0.09)	0.35
Carotid IMT (mm)	0.49 (0.46–0.49)	0.46 (0.43–0.49)	0.24

**Table 3:** Comparison of serum biomarkers according to metabolic health state in (a) children with normal weight (i.e., MHN vs. MUN) and (b) overweight/obese children (i.e., MHO vs. MUO).

Serum Biomarker	Children with Normal Weight			Overweight/Obese Children		
	MHN	MUN	*p* Value	MHO	MUO	*p* Value
**FGF21 (pg/mL)**	28.7 (11.0–87.3)	86.2 (28.2–107.9)	0.044	64.1 (8.1–120.4)	73.6 (48.7–118.2)	0.386
**Leptin (ng/mL)**	2.8 (1.0–10)	3.9 (2.2–5.8)	0.95	10.3 (5.8–13.8)	18.1 (10.7–31.5)	0.01
**IGFBP1 (ng/mL)**	3.0 (1.8–7.2)	2.3 (1.5–9.9)	0.84	2.6 (1.0–5.0)	1.5 (1.15–2.87)	0.403
**Adiponectin (μg/mL)**	8.6 (7.0–9.6)	8.0 (5.5–9.8)	0.64	8.5 (3.5–11.0)	7.3 (6.0–8.9)	0.68
**FGF21/Adiponectin (pg/μg)**	3.1 (1.5–8.7)	8.3 (3.7–13.3)	0.033	5.8 (0.9–33.7)	10.4 (5.4–19.5)	0.58

## Data Availability

The data that support the findings of this study are available from the corresponding author upon reasonable request.
